# Photobiomodulation, as additional treatment to traditional dressing of hard-to-heal venous leg ulcers, in frail elderly with municipality home healthcare

**DOI:** 10.1371/journal.pone.0274023

**Published:** 2022-09-15

**Authors:** Marianne Degerman, Micael Öhman, Bo C. Bertilson

**Affiliations:** 1 Department of Healthcare, Municipality of Skellefteå, Skellefteå, Västerbotten, Sweden; 2 Division of Wood Science and Engineering, Department of Engineering Sciences and Mathematics, Luleå University of Technology, Skellefteå, Västerbotten, Sweden; 3 Department of Neurobiology, Care Sciences and Society, Karolinska Institutet, Huddinge, Sweden; 4 Academic Primary Care Center, Stockholm Health Care Services (SLSO), Stockholm, Sweden; Massachusetts General Hospital, UNITED STATES

## Abstract

The main objectives of the study were to explore whether laser Photobiomodulation (PBM) in addition to traditional dressing of hard-to-heal venous leg ulcer, reduced healing time of the ulcer and if the duration of the ulcer before PBM impacted the treatment time with PBM to healing. The intervention group was frail, elderly patients with home healthcare in the municipality of Skellefteå, registered in the Swedish quality registry RiksSar for ulcer treatment with hard-to-heal venous leg ulcer. The control group with equivalent physical conditions was obtained from the same quality registry. Definition of hard-to-heal ulcer was six weeks duration or more. The PBM was performed two times per week with laser type infrared GaAs, 904nm, 60mW, and 700Hz, targeting lymphatic area and ulcer area. Laser type red visible, GaAllnp, 635nm, 75mW and 250Hz, targeting ulcer area. The intervention group treated with PBM in addition to traditional dressing healed significantly faster than the control group with a mean of 123 days (p = 0.0001). Duration of the ulcer before PBM did not impact the healing time. To conclude, the findings indicate that using PBM in addition to dressing may have multiple benefits on hard-to-heal venous leg ulcer, saving valuable time and resources for patients, healthcare providers, and institutions.

## Introduction

Wound healing is a dynamic process consisting of four continuous, overlapping, and precisely-programmed phases. Interruptions or prolongation in the process can lead to delayed or impaired wound healing and hard-to-heal ulcers (HHU). Factors affecting prevalence of HHU and wound healing include local and systemic factors. Local factors that directly influence the characteristics of the wound include oxygenation, infection, foreign body, and venous sufficiency. Systemic factors include the overall health or disease state of the individual that affect the ability to heal such as age, gender, ischemia, comorbidities, immunocompromised conditions, obesity, and nutrition to mention a few [1, 2]. Older adults are more likely to develop HHU than younger individuals. The effect of age and comorbidity on the effectiveness of existing and emerging treatments for HHU are unknown as older adults tend to be excluded from randomized clinical trials [1, 3].

There has been a rapid increase of the incidence and prevalence of HHU globally, with a majority of hard-to-heal leg ulcers of different aetiologies such as venous ulcers, pressure ulcers, and arterial ulcers [4–7]. Venous aetiology is the primary underlying factor in 70% of the HHU [8]. Long-term healing prognosis for HHU is poor, and worst for venous leg ulcers (VLU) [9]. Recurrence of VLU after healing 60–70% and the highest rate of recurrence within the first three months after healing [8, 10–12]. HHU have considerable economic and care resource impacts on the healthcare system, especially in outpatient settings [13, 14]. Various studies stress the importance of a holistic assessment and risk factor reduction in addressing the complex issues of HHU, especially in older adults [1–5].

PBM delivered using low-intensity laser has been shown to have the potential to improve wound healing and reduce pain, inflammation, oedema, and to regenerate damaged tissue such as wounds, bones, and tendons. PBM is a light therapy of low power intensity with a non-thermal process in tissue. Studies have shown that PBM induces a photochemical reaction at the cell and tissue level that affects three of the wound healing phases: the inflammatory phase, the proliferative phase, and the remodelling phase, and allows wound sites to close more rapidly. In addition, PBM is a non-invasive treatment with no known negative side effects [15–22].

The Swedish national quality registry RiksSar for ulcer treatment includes patients with HHU of different aetiologies. In 2020, the registry had a total of 20,242 registrations: 35% made by specialist units of hospital care, 43% by primary healthcare centres, and 22% by municipality primary home healthcare. The registry was started in 2009 [23].

The objectives of the study were to explore:

1. If PBM, in addition to traditional dressing of hard-to-heal VLU in frail elderly patients in municipality home healthcare, improves healing time of the VLU compared to a control group receiving traditional dressing only.
2. The possible impact of duration of the VLU, before PBM, on healing time.

## Materials and methods

This is a retrospective study from the Swedish quality registry RiksSar for ulcer treatment. The study was approved by the Ethical committee of Medicine in Lund Sweden with number 2020–02194. A written consent was obtained from the participant or the legal representative in the case of impaired cognition in the intervention group, the details of consent were declared in the application to the Ethical committee.

**The intervention group** consisted of patients in the municipality of Skellefteå with hard-to-heal VLU registered in RiksSar and treated by the home healthcare department during April 2019 to September 2020. Inclusion criteria were frail elderly patients risk-assessed and treated according to the guidelines in the quality register Senior Alert [24]. Patients were continuously admitted to municipality home healthcare throughout the study.

Exclusion criteria were:

1. Non-improvement: no visible reduction of ulcer size after six weeks of PBM, and no qualitative measures of an active healing process. One VLU was excluded due to non-improvement.
2. Personal choice: patients’ personal decision to interrupt PBM. Four VLU´s were excluded due to personal choice.
3. Decease of the patient during the treatment period. Four VLU´s were excluded due to decease.
4. Sars Covid-19 pandemic: six VLU´s were excluded due to circumstances connected to the pandemic.

The VLU´s in the intervention group with exclusion criteria 2–4 showed healing in progress until exclusion. The remaining intervention group consisted of 34 VLU´s in 27 patients.

To enable a control group, the VLU registrations in the RiksSar registry were used. Table 1 show patients in the RiksSar registry stratified into four groups A, B, C, D, according to duration of VLU in days, also the respective number of VLU´s being in treatment, having resulted in amputation, deceased, or having healed. The number of healed VLU diminished with duration and the risk of decease was considerably higher in group D, duration of 490 days or more. Patients with status healed VLU were selected as control group for this study.

**Table 1:** The RiksSar registry stratified into four groups (A-D) according to duration of the venous leg ulcer (VLU), also the respective number of VLU´s being in treatment, having resulted in amputation, deceased or having healed.

Group	Duration of VLU (days)	Group characteristics	In treatment	Amputated	Decease	Healed	Total
A	0–123	Number	11	0	0	193	204
		Female %	27			61	
		Age mean	79			78	
B	124–230	Number	3	0	0	196	199
		Female %	33			68	
		Age mean	77			80	
C	231–490	Number	15	0	5	190	210
		Female %	53		0	68	
		Age mean	80		81	80	
D	>490	Number	121	4	84	107	316
		Female %	62		62	61	
		Age mean	79		85	79	
	Total	Number	150	4	89	686	929

**The control group** consisted of VLU dressed according to physicians’ orders until healing occurred. Data extracted were ulcer area and patient age in the same range as for the intervention group, as well as gender, comorbidity and presence of diabetes. A total of 639 VLU´s in 531 patients from RiksSar were included. 47 VLU´s were excluded.

A conscious choice was made to allow a difference between the group’s ulcer area and patient age to be able to preserve as many VLU´s as possible in the control group.

### Treatment procedure

Each VLU was treated according to physicians’ orders based on the national recommendations for VLU treatment [25]. During the study, the VLU was photographed every week and its length and width was measured every three weeks until healing was obtained.

The PBM treatment procedure and dosing parameters were based on clinical experience of the patient group. Each VLU was treated two times per week, with an interval of two and three days. PBM equipment dose was calibrated in accordance with the manufacturer’s instructions before the study, every six months during the study, and after the completed study. The treatment team consisted of four healthcare trained specialists in ulcer treatment, with supervised PBM training and were given written treatment procedure with guidelines. The ulcer was first cleaned with 9 mg/ml NaCl solution and then treated with both infrared and red PBM.

Infrared PBM was administered with a 904nm, 60mW, GaAs laser with pulse frequency of 700Hz to treat targeted lymphatic area and ulcer area. The PBM treatment started at shoulder/ neckline for 2 minutes, dose 2.4J/cm^2^ bilateral and then in the hollow of the knee for 2 minutes, dose 2.4 J/cm^2^. Intact skin close to the VLU was thereafter treated during 30 seconds per location above, below, and on each side of the VLU dose 0.6/cm^2^, with contact application technique. Finally, the VLU surface was treated at a distance of 1cm with projection application technique, for 2 minutes per position until the total surface was treated with a dose of 2.4 J/cm^2^.

Red visible PBM was administered with a 635nm, 75mW, GaAllnp laser with pulse frequency of 250Hz to the ulcer edges 30 seconds per position with a dose of 0.8 J /cm^2^ and stepwise moved 1cm between positions with contact application technique, until the total ulcer edge was treated. The VLU surface was treated at a distance of 1cm with projection application technique, for 2 minutes per position until the total surface was treated with a dose of 3.1 J/cm^2^. After the treatment with infrared and red PBM, the VLU was dressed according to physicians’ orders.

### Statistical analysis

Due to the heterogeneity of the intervention group, an individual control group for each of the 34 VLU´s in the intervention group was extracted from the control group.

VLU´s in the individual control group had the same or longer healing time, than the intervention VLU duration when PBM started. This resulted in a total of 34 individual control groups, all different in number of ulcers.

The 34 individual control groups showed a large difference in number of observations of healing time between each other. To eliminate the bias caused by these large differences in number, the subsequent analysis is based on the observed 34 median differences in time to heal between the intervention group and the individual control groups. The 34 median values were used as observations in a Wilcoxon signed rank test to analyze if there was a statistically significant difference, with alpha value set to 0.01. The null hypothesis was: There is no difference in healing time between the intervention group and the control group.

The possible impact of ulcer duration before PBM for healing time with PBM was statistically analyzed using Pairwise Correlation analysis and Mahalanobis Distances analysis of the sensitivity toward outliers.

## Results

### Characteristics of the intervention and the control group

Women accounted for 85% of the intervention group and 67% of the control group. Comorbidities in the intervention group were three diagnoses and two in the control group. Diabetes was found in 44% of the patients in the intervention group and in 21% of the patients in the control group. Table 2 shows the characteristics of the VLU´s and the patients in the intervention group and the control group as well as difference in healing time between the intervention group being treated with PBM and the controls.

**Table 2:** Characteristics of the venous leg ulcers (VLU´s) and the patients in the intervention group and the control group as well as difference in healing time between the intervention group being treated with Photobiomodulation (PBM) and the controls.

Intervention Group								Control Group							Mean difference in healing time (days)
VLU	Patient age (years)	Gender (Female/Male) (Female = F/Male = M)	Comorbidities (Count)	Diabetes (Yes/No)	VLU area (cm^2^)	VLU days before PBM	PBM time to healing (days)	Mean number of VLU	Mean patient age (years)	Share of Females (%)	Mean number of comorbidities	Diabetes (%)	Mean VLU area (cm^2^)	Mean healing time (days)	
**01**	75	F	3	N	5	42	7	639	80	67	2	21	44	197	148
**02**	75	F	3	N	1	42	33	639	80	67	2	21	44	197	122
**03**	97	F	3	N	2	49	28	628	80	67	2	21	4	201	124
**04**	90	F	2	N	6	63	6	618	80	67	2	21	44	204	135
**05**	75	F	2	N	4	77	84	580	80	67	2	21	4	224	63
**06**	86	F	3	N	2	112	28	491	81	67	2	21	4	257	117
**07**	94	F	2	N	2	112	57	491	81	67	2	21	4	257	88
**08**	78	F	5	Y	1	119	79	474	81	67	2	22	44	266	68
**09**	84	F	3	N	30	140	140	436	81	67	2	22	44	289	9
**10**	91	M	3	Y	9	154	28	409	81	67	2	22	55	295	113
**11**	69	M	3	N	13	161	206	395	81	67	2	23	55	303	-64
**12**	69	M	3	N	56	161	148	395	81	67	2	23	55	303	-6
**13**	85	F	2	N	2	161	25	395	81	67	2	23	55	303	117
**14**	83	F	4	Y	9	168	54	382	81	67	2	23	55	310	88
**15**	83	F	4	Y	10	168	61	382	81	67	2	23	55	310	81
**16**	83	F	4	Y	5	168	61	382	81	67	2	23	55	310	81
**17**	85	F	3	N	4	182	26	354	81	66	2	23	55	333	125
**18**	85	F	3	N	8	182	35	354	81	66	2	23	55	333	116
**19**	85	F	3	N	1	182	35	354	81	66	2	23	55	333	116
**20**	85	F	3	N	3	259	37	245	81	65	3	23	5	420	124
**21**	91	F	2	N	1	448	35	107	82	64	3	16	55	692	209
**22**	83	F	5	N	11	630	50	59	82	64	2	14	5	977	297
**23**	90	F	5	N	7	1953	313	8	83	75	3	13	222	2797	531
**24**	65	M	2	Y	11	1022	49	26	83	62	2	19	10	1604	533
**25**	62	F	2	Y	4	56	105	623	80	67	2	21	44	203	42
**26**	62	F	2	Y	200	301	91	199	82	64	3	22	5	474	82
**27**	62	F	2	Y	150	301	147	199	82	64	3	22	5	474	26
**28**	82	F	3	Y	28	70	14	599	80	67	2	21	4	210	126
**29**	73	F	4	Y	20	63	56	618	80	67	2	21	44	204	85
**30**	73	F	4	Y	9	56	14	623	80	67	2	21	44	203	133
**31**	90	M	3	Y	12	42	14	639	80	67	2	21	44	197	141
**32**	87	F	3	N	9	49	42	628	80	67	2	21	4	201	110
**33**	88	F	4	Y	6	322	119	185	82	63	3	23	5	502	61
**34**	88	F	4	Y	3	140	112	436	81	67	2	22	4	289	37

Median age in the intervention group was 84 years and in the control group 80 years. The ulcer area in the intervention group was a median of 4 cm^2^ and in the control group 7 cm^2^. Table 3 shows the patient age and ulcer area distribution in the intervention group and the control group, with mean age, mean area, and its standard deviation and number of observations per category.

**Table 3:** Patient age and ulcer area distribution in the intervention group (IG) and the control group (CG).

		Quantiles							
	Level	Min.	25%	Median	75%	Max	Mean	Std Dev	No obs
Age	IG	60	74	84	87	97	80	10	34
(years)									
Age	CG	68	74	80	85	96	80	0.3	639
(years)									
Ulcer Area	IG	1	3	7	11	200	19	42	34
(cm^2^)									
Ulcer Area	CG	1	2	4	10	56	9	11	639
(cm^2^)									

Std Dev = Standard deviation, No obs. = Number of observations.

Distribution of numbers of VLU´s in the 34 individual control groups: median number was 402 VLU´s and mean number was 411 VLU´s per individual control group. S1 Fig.

### Healing time of VLU in the intervention and the control group

Healing time of the 34 hard-to-heal VLU´s in the intervention group receiving PBM was reduced between 66 and 180 days, with a mean of 123 days compared to the VLU´s in the control group receiving traditional dressing (p = 0.0001). S2 Fig.

### Possible impact of duration of the VLU´s, before PBM, on healing time

Duration of the VLU´s before PBM, did not affect the PBM healing time.

**Fig 1:**
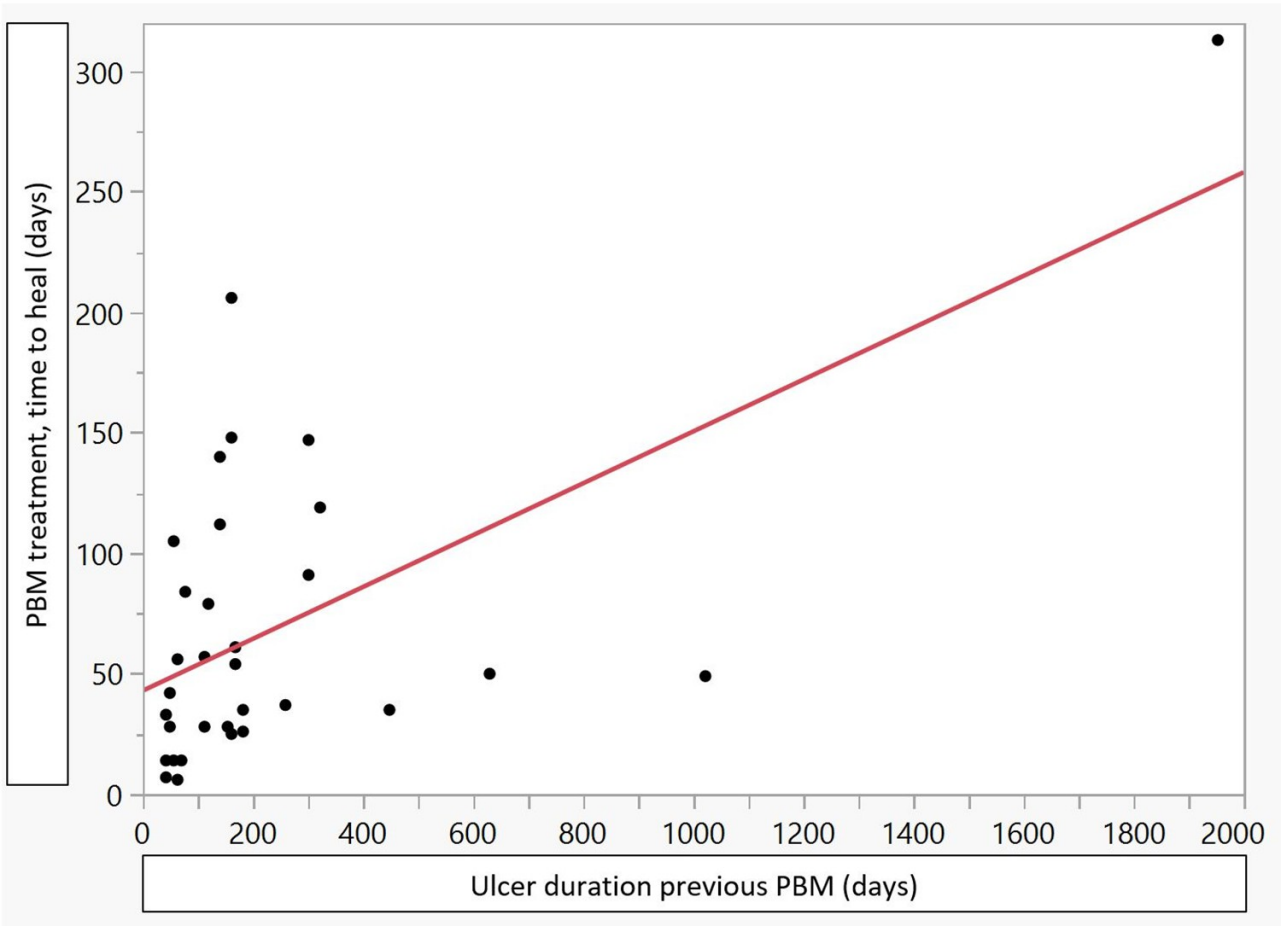
Dependence between venous leg ulcer (VLU) duration in days before photobiomodulation (PBM) and PBM time in days to heal VLU and linear correlation, 34 observations.

The observed dependence, in terms of linear correlation, between VLU duration before PBM for the healing time with PBM is ***r*** = 0.6. The diagram shows an absence of observations in a wide range between the most extreme observation and the rest. By excluding the three most extreme observations, identified by Mahalanobis distances, the linear correlation decreased to ***r*** = 0.23. Fig 1.

## Discussion

In this study, it was found that the hard-to-heal VLU´s of the patients in the intervention group which received the added PBM treatment, healed significantly faster than the VLU´s of the control group (p = 0.0001). The mean difference in healing time was 123 days. This pronounced difference indicates a great potential for the PBM treatment. The intervention group exhibited a higher level of frailty than the control group being older, having more comorbidities including a larger percentage of diabetes. In defiance of those poorer circumstances the hard-to-heal VLU´s in the intervention group healed at least 66 days faster than the VLU´s of the control group.

The possible impact of duration of the VLU´s before intervention with PBM was found to have a correlation of 0.6. However, the correlation was foremost caused by one single observation. By using the Mahalanobis Distances and excluding the three most extreme observations, the correlation decreased to 0.23. This indicates that the duration of the VLU before intervention with PBM treatment, may be regarded as a poor indication to predict treatment time with PBM. Previous perceptions and studies state diminishing probability of ulcer healing the longer the duration of the VLU [9]. The findings in this study suggest that PBM may provide a new approach in the treatment of hard-to-heal and long-term hard-to-heal VLU adding encouragement and a treatment tool for patients and professionals struggling with VLU treatment.

This study, in accordance with other studies of PBM treatment, found beneficial effects on ulcer healing [15–22]. However, the biological and biochemical effects and mechanisms of PBM are still under investigation [15–17]. Different PBM irradiation and wavelengths have been evaluated in various studies with the aim to optimize PBM treatment [18, 19]. PBM in the range of 390–685 nm have been identified to affect superficial tissue, and longer wavelengths 808–904 nm are identified to stimulate deep-seated tissues. Whether to use red or near infrared light or a combination of both is also under investigation. Studies have compared different wavelengths or chosen to use a specific light to treat ulcers, and there is no agreement on the optimal application [18–22]. In this study, a combination of red and infrared light was used. The aim was to stimulate growth factors in the superficial tissue and increase circulation in deep tissue. In the home healthcare environment treatment time is a key. The risk of a too low dose to reach a result of PBM was identified as a greater risk than the risk of a too high dose that in other studies has shown to be less effective [15]. The treatment procedure and PBM dose in this study was standardized to be adequate, simple, and accessible to perform in the home healthcare environment. Our findings indicate that the combined treatment was successful.

A strength in this study was the possibility to compare healing time and other data with the large Swedish registry RiksSar and to tailor individual control groups for every VLU treated with PBM, resulting in a mean of 411 comparisons per treated VLU. Another strength is the possibility to follow the study cohort in home healthcare until healing.

Limitations of our study include the lack of a randomization of different treatment doses including placebo. Another limitation was the lack of laboratory tests to ascertain biochemical healing processes.

Further studies are needed to determine if there are optimal PBM treatment doses for different patient categories, and also investigate the durability of PBM in VLU treatment and biological reactions in the frail elderly body and the VLU´s.

## Conclusions

Regardless of the frailty of the intervention group, PBM, in addition to traditional dressing, significantly improved healing time of hard-to-heal VLU´s with a mean of 123 days, compared to the control group.

The duration of the VLU before initiating PBM treatment did not affect healing time with PBM. Incorporating PBM into mainstream VLU treatment may significantly change outcomes of treatment. Shortening ulcer duration and promoting healing, including long-term hard-to-heal VLU saves time and resources, for patients, professionals, and healthcare institutions.

## Supporting information

S1 Fig Distribution of numbers of VLU in the 34 individual control groups. Distribution of numbers of VLU in the individual control groups. X-axis = number of control group VLU. Y-axis number of individual control groups.
(PDF)

S2 Fig Healing time of VLU in the intervention and control group. Distribution median difference in healing time intervention and control group VLU.
(PDF)

## References

[1] Guo S, DiPietro LA. Factors Affecting Wound Healing. J Dent Res. 2010;89(3):219–229. doi: 10.1177/0022034509359125 20139336 PMC2903966

[2] Vieira C, Araújo TM. Prevalence and factors associated with chronic wounds in older adults in primary care. Rev Esc Enferm USP. 2018;52:e03415. doi: 10.1590/S1980-220X2017051303415 30569961

[3] Gould L, Abadir P, Brem H, Carter M, Conner-Kerr T, Davidson J, et al. Chronic Wound Repair and Healing in Older Adults: Current Status and Future Research. JAGS. 2015;63:427–438. doi: 10.1111/jgs.13332 25753048 PMC4582412

[4] Martinengo L, Olsson M, Bajpai R, Soljak M, Upton Z, Schmidtchen A, et al. Prevalence of chronic wounds in the general population: systematic review and meta-analysis of observational studies. Annals of Epidemiology 2019;29:8–15. doi: 10.1016/j.annepidem.2018.10.005 30497932

[5] Jeyaraman K, Berhane T, Hamilton M, Chandra AP, Falhammar H. Mortality in patients with diabetic foot ulcer: a retrospective study of 513 cases from a single Centre in the Northern Territory of Australia. BMC Endocrine Disorders 2019;19:1. doi: 10.1186/s12902-018-0327-2 30606164 PMC6318899

[6] Chan B, Cadarette S, Wodchis W, Wong J, Mittmann N, Krahn M. Cost-of-illness studies in chronic ulcers: a systematic review. J Wound Care 2017;26:S4–14. doi: 10.12968/jowc.2017.26.Sup4.S4 28379102

[7] Heyer K, Herberger K, Protz K, Glaeske G, Augustin M. Epidemiology of chronic wounds in Germany: analysis of statutory health insurance data. Wound Repair Regen 2016;24:434–42. doi: 10.1111/wrr.12387 26609788

[8] Abbade LPF, Lastória S. Venous ulcer: epidemiology, physiopathology, diagnosis and treatment. Int J Dermatol. 2005;44:449–456. doi: 10.1111/j.1365-4632.2004.02456.x 15941430

[9] Nelzén O, Bergqvist D, Lindhagen A. Long-term Prognosis for Patients with Cronic Leg Ulcers: a Prospective Cohort Study. Eur J Vasc Endovasc Surg.1997;13:500–508. 9166274 10.1016/s1078-5884(97)80179-7

[10] Finlayson K, Parker C, Miller C, Gibb M, Kapp S, Ogrin R, et al. Predicting the likelihood of venous leg ulcer recurrence: The diagnostic accuracy of a newly developed risk assessment tool. Int Wound J. 2018;15:686–694. doi: 10.1111/iwj.12911 29536629 PMC7949606

[11] Moffatt CJ, Dorman MC. Recurrence of leg ulcers within a community ulcer service. J Wound Care 1995;4:57–61. doi: 10.12968/jowc.1995.4.2.57 7600337

[12] Finlayson K, Edwards H, Courtney M. Factors associated with recurrence of venous leg ulcers. Int J Nurs Stud. 2009;46:1071–1078. 19185862 10.1016/j.ijnurstu.2008.12.012

[13] Nussbaum S, Carter M, Fife C, DaVanzo J, Haught R, Nusgart M, et al. An Economic Evaluation of the Impact, Cost, and Medicare Policy Implications of Chronic Nonhealing Wounds. Value In Health 2018;21:27–32. doi: 10.1016/j.jval.2017.07.007 29304937

[14] Guest JF, Vowden K, Vowden P. The health economic burden that acute and cronic wounds impose on an average clinical commissioning group/health board in the UK. J Wound Care 2017;26:292–303. 28598761 10.12968/jowc.2017.26.6.292

[15] Chung H, Dai T, Sharma S, Huang Y, Carroll J, Hamblin R. The Nuts and Bolts of Low-level Laser (Light) Therapy. Annals of Biomedical Engineering 2012;40:516–533. doi: 10.1007/s10439-011-0454-7 22045511 PMC3288797

[16] Hamblin M. Mechanisms and Mitochondrial Redox Signaling in Photobiomodulation. J Photochemistry & Photobiology 2018;94:199–212. doi: 10.1111/php.12864 29164625 PMC5844808

[17] Tsai S, Hamblin M. Biological effects and medical applications of infrared radiation. J Photochemistry & Biology 2017;170:197–207. doi: 10.1016/j.jphotobiol.2017.04.014 28441605 PMC5505738

[18] Taradaj J, Shay B, Dymarek R, Sopel M, Walewicz K, Beeckman D, et al. Effect of laser therapy on expression of angio- and fibrogenic factors, and cytokine concentrations during the healing process of human pressure ulcers. Int. J Med. Sci. 2018;15(11):1105–1112. doi: 10.7150/ijms.25651 30123047 PMC6097266

[19] Avci P, Gupta A, Sadasivam M, Vecchio D, Pam Z, Pam N, et al. Low-Level Laser (Light) Therapy (LLLT) in Skin: Stimulating, Healing, Restoring. Semin Cutan Med Surg. 2013;32:41–52. 24049929 PMC4126803

[20] Santos C, Rocha R, Hazime F, Cardoso V. A Systematic Review and Meta-Analysis of the Effects of Low-Level Laser Therapy in the Treatment of Diabetic Foot Ulcers. Int. J Lower Extremity Wounds 2020;0:1–10. doi: 10.1177/1534734620914439 32394760

[21] Feitosa M, Carvalho A, Feitosa V, Coelho I, Oliveira R, Arisawa E. Effects of the Low-Level Laser Therapy (LLLT) in the process of healing diabetic foot ulcers. Acta Cirurgica Brasileira 2015;30:852–857. doi: 10.1590/S0102-865020150120000010 26735058

[22] Kuffler. Photobiomodulation in promoting wound healing: a review. Regen Med. 2016;11:107–122. doi: 10.2217/rme.15.82 26681143

[23] RiksSår, the Swedish national quality Registry for Ulcer Treatment. 2021 Jun 22 [cited 2 September 2022]. Available from: (rikssar.se).

[24] Senior Alert 2021 May 31 [cited 2 September 2022]. Available from: English—Senior alert.

[25] Vårdhandboken. 2020 Jul 16 [cited 2 September 2022]. Available from: Vardhandboken.se.

