# Supplementary material for: Photobiomodulation, as additional treatment to traditional dressing of hard-to-heal venous leg ulcers, in frail elderly with municipality home healthcare
Source: PLoS One. 2022 Sep 15;17(9):e0274023. doi: 10.1371/journal.pone.0274023 (PMC9477261; doi:10.1371/journal.pone.0274023)
Supplement: S1 Fig — Distribution of numbers of VLU in the individual control groups. X-axis = number of control group VLU. Y-axis number of individual control groups. (PDF) [file pone.0274023.s001.pdf]

S1 Fig.

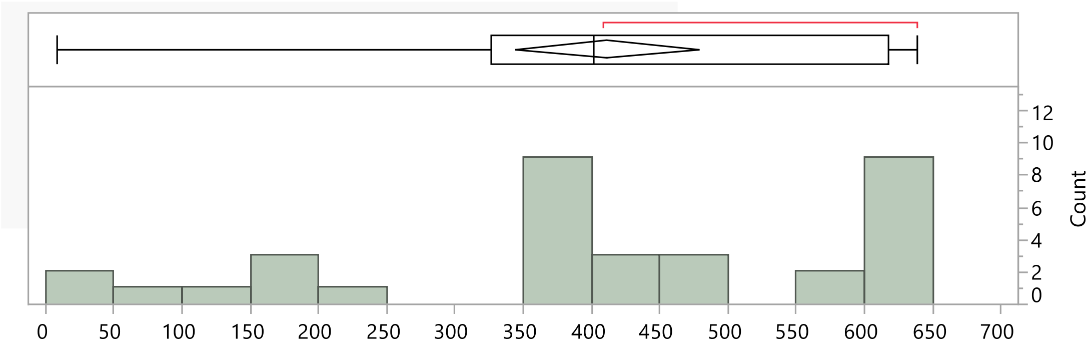

Fig. 1. Distribution of numbers of VLU in the individual control groups. X-axis = number of control VLU. Y-axis number of individual control groups.

Quantiles

100.0%	maximum	639
99.5%		639
97.5%		639
90.0%		633,5
75.0%	quartile	618
50.0%	median	402
25.0%	quartile	326,75
10.0%		83
2.5%		8
0.5%		8
0.0%	minimum	8

Summary Statistics

Mean	411,52941
Std Dev	191,97115
Std Err Mean	32,922781
Upper 99% Mean	501,51648
Lower 99% Mean	321,54234
N	34

Confidence Intervals

Parameter	Estimate	Lower CI	Upper CI	1-Alpha
Mean	411,5294	321,5423	501,5165	0,990
Std Dev	191,9712	145,2443	277,303	0,990
