# Supplementary material for: Photobiomodulation, as additional treatment to traditional dressing of hard-to-heal venous leg ulcers, in frail elderly with municipality home healthcare
Source: PLoS One. 2022 Sep 15;17(9):e0274023. doi: 10.1371/journal.pone.0274023 (PMC9477261; doi:10.1371/journal.pone.0274023)
Supplement: S2 Fig — Distribution median difference in healing time intervention and control group VLU. (PDF) [file pone.0274023.s002.pdf]

S2 Fig.

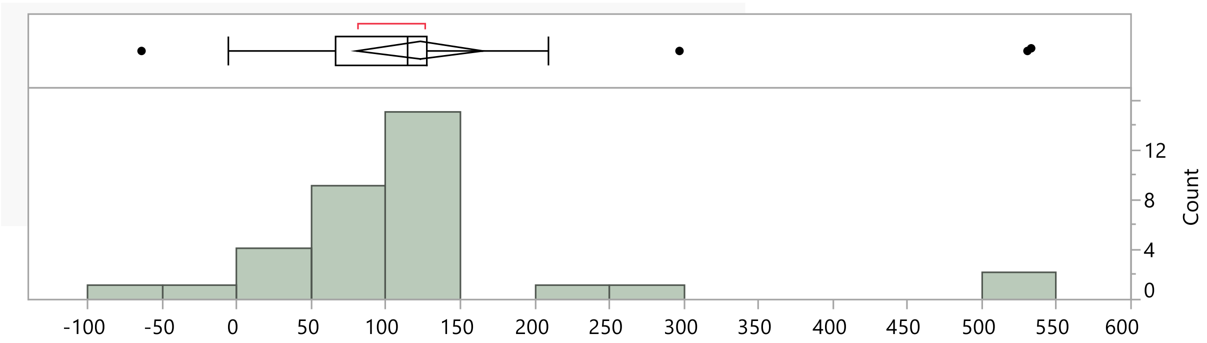


Fig. 2. Distribution median difference in healing time intervention and control group VLU. Y-axis = intervention group VLU. X-axis = difference in healing time, number of days.

## Quantiles

| | | |
|---|---|---|
| 100.0% | maximum | 533 |
| 99.5% | | 533 |
| 97.5% | | 533 |
| 90.0% | | 253 |
| 75.0% | quartile | 127,75 |
| 50.0% | median | 114,25 |
| 25.0% | quartile | 66,25 |
| 10.0% | | 17,5 |
| 2.5% | | -64 |
| 0.5% | | -64 |
| 0.0% | minimum | -64 |

## Summary Statistics

| | |
|---|---|
| Mean | 122,76471 |
| Std Dev | 120,75183 |
| Std Err Mean | 20,708767 |
| Upper 99% Mean | 179,3675 |
| Lower 99% Mean | 66,161916 |
| N | 34 |

## Confidence Intervals

| Parameter | Estimate | Lower CI | Upper CI | 1-Alpha |
|---|---|---|---|---|
| Mean | 122,7647 | 66,16192 | 179,3675 | 0,990 |
| Std Dev | 120,7518 | 91,36017 | 174,4264 | 0,990 |
